# Aerosol Measurement Degradation in Low-Cost Particle Sensors Using Laboratory Calibration and Field Validation

**DOI:** 10.3390/toxics11010056

**Published:** 2023-01-06

**Authors:** Angela Peck, Rodney G. Handy, Darrah K. Sleeth, Camie Schaefer, Yue Zhang, Leon F. Pahler, Joemy Ramsay, Scott C. Collingwood

**Affiliations:** 1 Occupational and Environmental Health, Department of Family and Preventive Medicine, University of Utah, Salt Lake City, UT 84108, USA; 2 Department of Family and Preventive Medicine, University of Utah, Salt Lake City, UT 84108, USA; 3 Department of Internal Medicine, University of Utah, Salt Lake City, UT 84108, USA; 4 Department of Pediatrics, University of Utah, Salt Lake City, UT 84108, USA

**Keywords:** particulate matter, low-cost sensors, exposure assessment, sensor degradation, citizen science

## Abstract

Increasing concern over air pollution has led to the development of low-cost sensors suitable for wide-scale deployment and use by citizen scientists. This project investigated the AirU low-cost particle sensor using two methods: (1) a comparison of pre- and post-deployment calibration equations for 24 devices following use in a field study, and (2) an in-home comparison between 3 AirUs and a reference instrument, the GRIMM 1.109. While differences (and therefore some sensor degradation) were found in the pre- and post-calibration equation comparison, absolute value changes were small and unlikely to affect the quality of results. Comparison tests found that while the AirU did tend to underestimate minimum and overestimate maximum concentrations of particulate matter, ~88% of results fell within ±1 μg/m^3^ of the GRIMM. While these tests confirm that low-cost sensors such as the AirU do experience some sensor degradation over multiple months of use, they remain a valuable tool for exposure assessment studies. Further work is needed to examine AirU performance in different environments for a comprehensive survey of capability, as well as to determine the source of sensor degradation.

## 1. Introduction

In areas that experience sustained and/or severe air pollution episodes, the ability to monitor levels on a day-to-day basis has increasingly become a priority for both public health professionals and the general population [1]. One such area in need of daily monitoring is the Salt Lake Valley in Utah, where a high population density and unique geographical makeup (i.e., surrounding mountain ranges that can “trap” polluted air during temperature inversion episodes) contribute to high exposure to air contaminants, including particulate matter (PM) sized 2.5 μm or less (PM_2.5_) [2]. Over the past ten years, local concentration levels have regularly exceeded the PM_2.5_ limits set by the United States Environmental Protection Agency (EPA) [3].

Research suggests that chronic exposure in this vein may have profound health impacts, including increased rates of cardiovascular disease, pulmonary disease, and respiratory outcomes such as aggravated asthma and decreased lung function, all of which may culminate in early mortality [4,5,6,7,8]. Vulnerable populations, such as children, the chronically ill, and the elderly are particularly at risk due to increased particulate pollution [9,10,11,12,13] PM_2.5_, which results primarily from combustion activities (e.g., traffic, cigarette smoking, coal burning), poses a particularly high health risk [14]. The small size of PM_2.5_ results in the particles remaining in the air longer, thereby increasing the likelihood of inhalation. In addition, particles of this size penetrate deeply into the respiratory system and bloodstream, creating long-term damage to the respiratory system as well as systemic effects [7,15].

The effects of chronic PM exposure have been long observed in residents of the Salt Lake Valley. Historically, higher amounts of PM in the Salt Lake Valley have been associated with greater numbers of hospital admissions, with one recent study estimating that at least 200 pneumonia cases per year in Utah can be linked to episodes of increased PM pollution (III 1991). Horne et al. (2018) reported that following exposure to high levels of PM, patients were 16% more likely to suffer a heart attack or chest pain ([16,17], while Leiser et al. (2019) associated air pollution with increased rates of spontaneous pregnancy loss [18] Similarly, from the 2019 American Thoracic Society report, Salt Lake City would experience 46 fewer deaths annually if PM_2.5_ air quality goals were attained [19]. Per Utah clinicians and researchers, there are numerous other consequences of Salt Lake City PM_2.5_ air pollution, including an excess of 50,000 lost work and school days annually due to air pollution-related health issues [20,21]. Access to air quality monitoring can help implement preventive actions, such as limiting recess and outdoor activities on poor air quality days and use of household air filters [22,23]. Consequently, researchers, government officials, and individual citizens have worked to develop extensive air quality monitoring programs. Among these is the Environmental Influences on Child Health Outcomes (ECHO) multi-site project, a National Institutes of Health (NIH) funded program, which includes exposure assessment monitoring of ambient air quality in and around households of enrolled children (NIH).

In recent years, air quality monitoring initiatives such as these have begun to incorporate low-cost sensors useable in citizen science initiatives rather than reference-grade instrumentation suitable for laboratory research [24]. Low-cost sensors encompass a wide variety of brands and technology but are generally economical and consumer-friendly devices designed to monitor aspects of air pollution (including PM) (Lung). The University of Utah has developed an addition to the low-cost sensor market, an integrated air quality sensor named the AirU (Utah). These sensors have been deployed in multiple ongoing research studies, including at the University of Utah ECHO local study site.

The performance of low-cost sensors has been evaluated in several laboratory studies with good results including Dylos vs. TSI DustTrak [25]; Dylos vs. GRIMM [26] and Shinyei vs. TSI instruments [27,28,29] with moderate to strong correlations when compared to the reference instruments. A few studies have investigated the use of low-cost sensors to examine indoor air quality with moderate to good results when compared to reference instruments deployed in the same environment [30,31], and Hegde et al. [32] co-located various low-cost sensors with reference instruments demonstrating the utility of the low-cost sensors in characterizing the indoor air quality.

However, the long-term performance of low-cost sensors and sensor units m, such as the AirU has yet to be fully validated. Field research reveals that low-cost sensors can experience performance issues, including variability within units of the same sensor brand and differences between laboratory and field test performance [26,33,34]. Prior studies have suggested that sensor degradation of data accuracy over time needs to be further characterized to fully understand the potential and limitations of low-cost sensors [35,36] It is presently unknown how much time may pass before a sensor becomes inaccurate in its readings, and furthermore, it is not well understood what causes inaccuracy over time. At least one study has postulated that measurement degradation results from an accumulation of dust over the course of time [35] while another study examined relative humidity during performance periods to account for variation [37].

As low-cost sensors become increasingly integral to air quality monitoring, it is critical to validate sensor measurements in absolute and practical settings to ensure data quality [38]. The purpose of this study was to examine low-cost sensor performance using two metrics: (1) pre- and post-deployment calibration equations from laboratory tests, and (2) long-term residential field validation with reference-grade instrumentation for comparison. By comparing pre- and post-deployment calibration equations derived from laboratory tests we can investigate if the low-cost sensors underwent a measurement variation over time. As these low-cost units lack sophisticated pre-filters or even flow-rate monitoring of the sampled air moving through the sensor instrumentation, comparing pre- and post-deployment calibration equations will evaluate how these units performed over several months of continuous operation in homes. The long-term field study conducted in a single residential home with multiple low-cost units co-located with a reference instrument will provide an opportunity to compare the ability of the low-cost sensors to accurately estimate PM over the extended operation (time-series) in an indoor environment.

## 2. Methods

This project quantitatively evaluated the performance of the AirU by two means. First, pre-deployment calibration equations (henceforth referred to as “pre-calibration”) were compared to post-deployment calibration equations (henceforth referred to as “post-calibration”) following varying periods of deployment in the homes of participants voluntarily engaged in a longitudinal research study conducted in the Salt Lake Valley, Utah. Secondly, three AirU units not previously deployed were compared to a reference-grade instrument in a long-term field validation inside a single Salt Lake Valley household.

### 2.1. AirU Units

AirUs are integrated low-cost, Wi-Fi and GPS-enabled devices containing sensors for temperature, relative humidity, and particulate matter (PM); the particle counter used inside the AirU is the Beijing Plantower Company’s PMS 3003 sensor. The PMS 3003 model uses a fan to draw air into its chamber, which is then exposed to a laser estimated to be 650 ± 10 nm with a Lambda 35 spectrometer. Using a MIE theory for the processor, the light scattering sizes particles into three separate bins: PM1, PM_2.5_, and PM10 ([39]. The PMS 3003 model can detect particles in the range of 0.3 μm to 10 μm [26,40]. The limit of detection (LOD) for this device, as reported by others, is ~5 μg/m^3^ [26,41], which, in practice, can be considered a limit of quantification (LOQ). The PMS 3003 sensor was placed in an injection-molded plastic housing that allows for the inlet fan to draw air through it and a USB charging cable to be plugged into power the sensor.

### 2.2. Pre- and Post-Calibration Equations

In order to assess sensor differences between baseline and following a period of extended use, a subset of AirU sensors (n = 24) were analyzed that were deployed as part of the University of Utah’s ECHO study site in the Salt Lake Valley. These sensors were in the field for varying lengths of time over the period of January 2018–October 2018. Each sensor was calibrated prior to deployment and at the end of its deployment, which occurred at varying times throughout the research period for different sensors.

A calibration equation is a linear regression equation that relates the concentration measured by the AirU to the concentration measured by a reference instrument. The calibration protocol was identical for pre- (i.e., prior to field use) and post-deployment calibrations. In this way, sensors were re-calibrated prior to returning to field use. All post-deployment calibrations and analyses for this research period took place from March 2018 to October 2018. This process is described in detail in Kelly et al. (2017); relevant methods are briefly described below.

Each Plantower PMS 3003 sensor was evaluated in a cylindrical wind tunnel calibration chamber located in the Merrill Engineering Building on the University of Utah campus [42]. The chamber can hold 8 sensors and has an entry hole for the reference instrument, which was a Dust Trak II Aerosol Monitor 8530. This instrument is capable of measuring PM1, PM_2.5_, respirable, and PM10 size fractions (TSI). The Dust Trak was selected as a reference instrument in part due to its use of a sheath air system to isolate the aerosol, which keeps the optics clean for improved reliability. It also possesses automatic zeroing by the instrument, which minimizes the effect of zero drift [8]. The wind tunnel calibration chamber and method were created specifically to calibrate the Plantower PMS 3003 sensors, and this standard operating procedure was followed [42].

Per the calibration process developed by Sayahi et al. [42], ammonium nitrate was aerosolized and entrained in air that had passed through a high-efficiency particulate air (HEPA) filter, which was then evenly distributed throughout the chamber [2]. This compound was used because it was the most common particle type found in filters from sensors used during Utah winter inversions (Engineering). Chamber aerosol concentrations, as measured by the Dust Trak, of 0, 50, 100, 150, 200, and 250 μg/m^3^ were obtained by allowing more particles to enter the HEPA-filtered air. When each aerosol concentration was attained and sustained, as indicated by the Dust Trak, at least 10 min of continuous measurement was recorded before adjusting the aerosol to the next concentration. The entire calibration process lasts approximately 1.5 h, with additional time required for measurement data retrieval and calibration equation generation.

The data were then extracted, via SD card for the AirU and through USB for the Dust Trak, and converted to Microsoft Excel files (Microsoft Corporation, Redmond, WA, USA). The data were averaged into 1-min increments for both the AirUs and the Dust Trak, and converted to μg/m^3^ where necessary. It was also necessary to apply a correction factor to the Dustrak for comparison to the AirU, based on research that indicates the Plantower sensor (used in the AirU) tends to underestimate concentrations [41,43]. Only data obtained during stable concentration levels were used for generating the calibration equation. The data for each individual sensor were compared against the Dust Trak data to obtain a linear regression equation. That equation was then used to adjust the raw AirU data to more accurately reflect estimated air concentrations.

### 2.3. Statistical Analysis

All statistical analysis for this portion of the study was performed in Microsoft Excel and SAS (SAS Institute, Cary, NC, USA). Due to non-normally distributed data, a Wilcoxon signed-rank test was performed on the parameters of the pre- and post-calibration equations for each individual sensor, including Y-intercept, slope, and R^2^, to determine if there were any statistical differences from the baseline calibration. The percentage differences for the Y-intercept, slope, and R^2^ were averaged for each of the 24 AirUs and then categorized into deployment time ranges of <3 months, 3–5 months, and >5 months.

### 2.4. Field Validation

In addition to the pre- and post-calibration analysis described above, a field validation test was conducted to examine differences in performance between the AirUs and reference-grade instrumentation over time. Three AirUs that had not been previously deployed were calibrated and used alongside a GRIMM Aerosol Monitor 1.109 (GRIMM Aerosol Technik GmbH & Co. KG, Ainring, Germany). These instruments were placed in a residential home between 8 November 2018 and 31 March 2019. The GRIMM is a portable laser aerosol spectrometer designed for continuous measurement of airborne particle concentration as well as particle size distribution (with 31 discrete size bins from 0.25 μm to 32 μm). This instrument also has a backup filter that collects all sampled material and can be used for further analysis if desired [44]. The GRIMM accomplishes its measurements with a light scattering method for single particles using a semiconductor laser, allowing it to detect particles as small as 0.25 μm [44].

**Figure 1:**
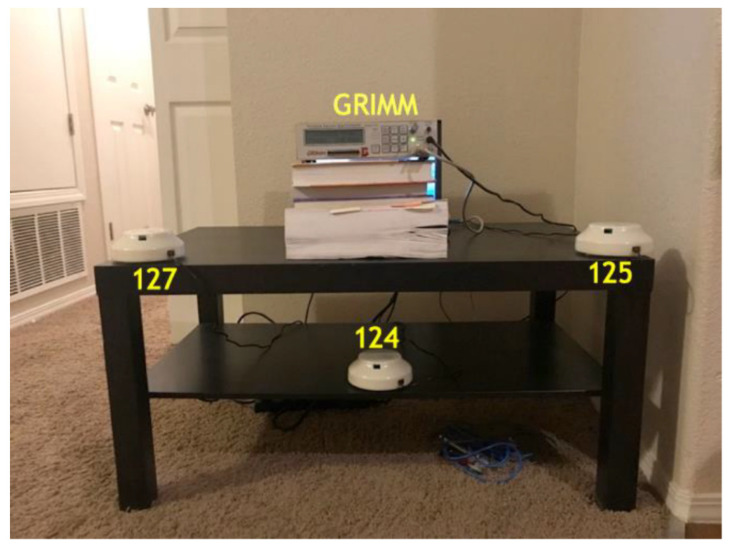
Photograph of AirU and GRIMM configuration for comparison in residential home. The GRIMM (labeled) was placed in an elevated position and the three AirUs (124, 125, 127) were triangulated around it.

All four instruments were placed inside a living room, which was chosen because it was the location of most day-to-day activities. The sensors were placed in a triangular configuration, with the GRIMM in the middle (see Figure 1) of the AirUs. Due to ventilation patterns and to prevent devices from interfering with each other’s air flow, sensors were placed apart from one another in a triangular configuration (ACGIH 1998). The GRIMM uploaded data every minute onto a laptop computer, while the AirUs stored data on their respective SD cards, which were later extracted.

The field validation was performed during the winter inversion season for 144 days. There was a self-test at start-up and twice during the trial. For those tests, the GRIMM was turned off, which initiated a self-check taking 39 s before it began to continue taking measurements. As the AirU began recording 15 s after powering on, the AirUs were powered off and then back on 24 s after the GRIMM was initiated in order to synchronize the time stamps.

### 2.5. Statistical Analysis

All analyses for the field validation were performed in R (Bell Laboratories, Murray Hill, NJ, USA). Maximum, minimum, mean, and median concentration measurements for the three AirUs and the GRIMM were calculated with no adjustment factors applied.Scatterplots were generated to examine the fit of the unadjusted AirU data with GRIMM data to establish how well the AirU could predict the true air concentration, as provided by the GRIMM. Time series were also created (using unadjusted data) to examine any deviation of the AirUs compared to the GRIMM over time.

For further comparison with the GRIMM, a linear regression model was created using the relationship between the PM_2.5_ concentrations measured by the GRIMM and the AirUs. 30 days of data were used to generate a calibration equation which was then applied to the following 30 days of data. This process was carried out sequentially for all data. This represents an on-site field calibration, which was deemed to be sufficient for adjusting data in lieu of using a calibration chamber [26]. Changes in the parameters from each of these field calibration equations (R^2^, Y-intercept, and slope) were also evaluated to investigate if these components changed over the course of the field validation test. In this way, the analysis examined calibration changes from month to month. Additional analyses were carried out on these adjusted AirU data to identify (a) how often the GRIMM and AirU were both measuring below the LOQ and (b) to how often the AirU and GRIMM measurements were within ±1 μg/m^3^.

## 3. Results

### 3.1. Pre- and Post-Calibration Equations

A Wilcoxon Signed Rank test found that changes between pre- and post-equations were not statistically different in terms of slope (*p* = 0.1753), but both the R^2^ (*p* = 0.0187), and Y-intercept (*p* ≤ 0.0001) were statistically different. Differences between regression parameters stratified based on the amount of time each AirU was deployed in the field can be seen in Table 1. There were no significant associations between time in the field and changes to calibration equation parameters.

**Figure 2:**
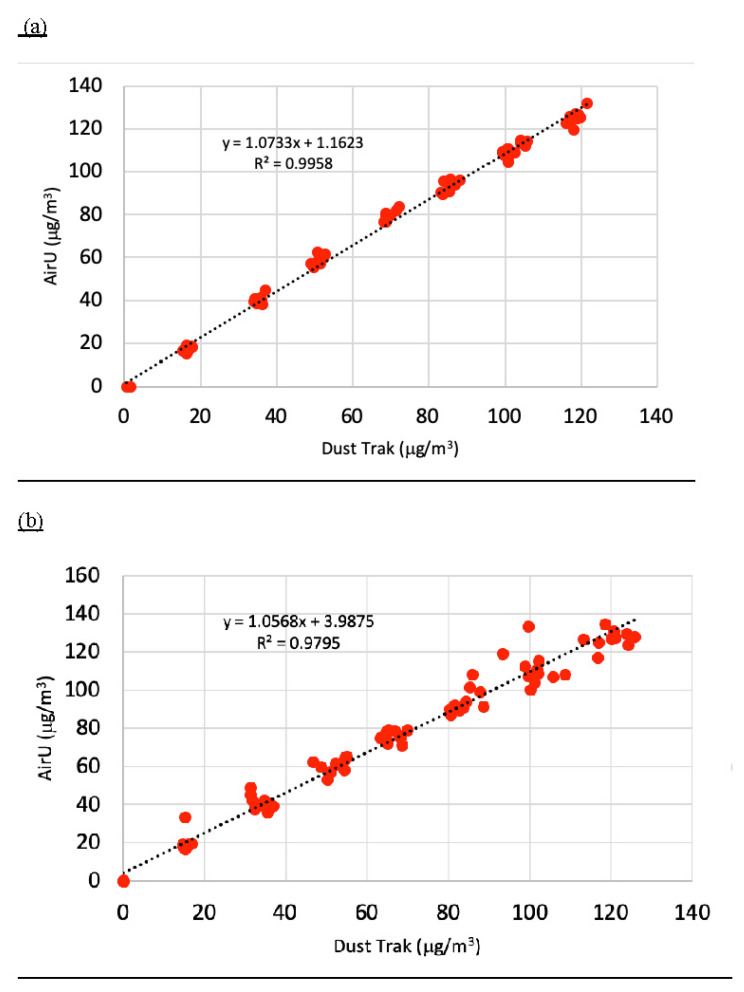
Changes in calibration equation in a single represent AirU. (**a**) is the pre-calibration equation and (**b**) is a post-calibration equation after 189 days in field.

An example of one of the 24 deployed AirU’s pre- and post-calibration line and regression equation are depicted in Figure 2. This particular sensor was deployed for 189 days and reflects trends in the overall findings. Overall, for the 24 sensors which were deployed for more than five months, the actual average change for the Y-intercept was 512%, 11% for the slope, and 1% for the R^2^.

### 3.2. GRIMM and AirUs Field Validation

Some AirU data from the field validation were excluded due to a hardware/software issue that caused the device to record a PM_2.5_ concentration of −1.0 μg/m^3^. Additional data were missing due to a problem with the SD card (i.e., it was not fully inserted) for one of the AirUs (Sensor 125). Although this was discovered and corrected during the quality control check in December, approximately one month of data are missing from the results. Finally, the limit of quantification (LOQ) for the AirU is ~5 μg/m^3^ [35,43] and each AirU had a majority of their measurements below this limit: AirU 124 (83% < LOQ), AirU 125 (89% < LOQ), and AirU 127 (80% < LOQ). However, the AirU did provide concentration values below this LOQ, so those data were still included in the analysis.

Prior to application of a correction factor, the raw maximum measurement for the three AirUs tended to be around four times the GRIMM measurement (see Table 2). Comparison of raw data, using AirU R^2^ as an indicator of the strength of the relationship with the GRIMM, revealed R^2^ values of 0.82 (Sensor 124), 0.97 (Sensor 125), and 0.935 (Sensor 127). See Figure S1 in the Supplementary Materials for comparisons between individual sensors and the GRIMM. While the graphs shown in Figure S1 of the Supplementary Materials are not of optimal quality, the reader should keep in mind that they are included to just show general clustering, trend, variability, and slope.

**Figure 3:**
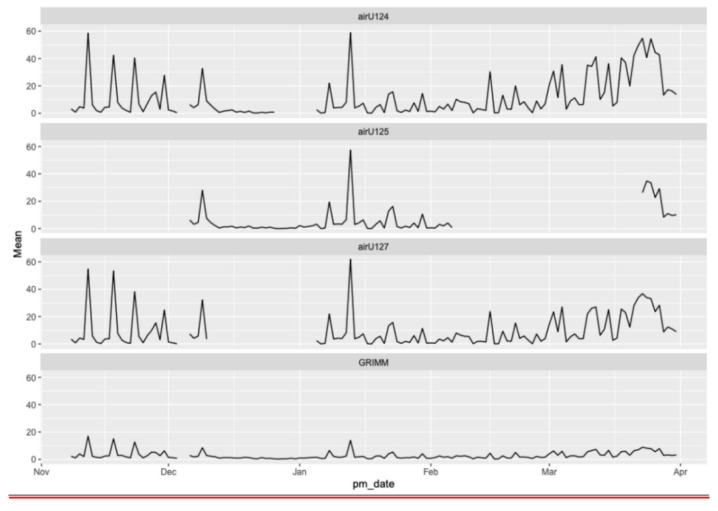
Time Series depicting daily average PM counts (in µg/m^3^) for AirU/Grimm comparison.

Figure 3 depicts the time series of the three AirUs (unadjusted measurements) and the GRIMM measurements over the study period. Of note are the empty areas in the series, which represent data gaps due to AirU circuit board errors. The graphical depiction of the time series data from the instruments PM_2.5_ exposure estimates visually demonstrates large agreement between the reference and low-cost monitors measurements when co-located in the same home environment. Periods of low-level PM_2.5_ aerosol are appropriately recorded by all the data logging instruments. Moreover, pollution events, where PM_2.5_ aerosol measurements increase are also captured by all the instruments alike. When examining the time series data of the co-located GRIMM and AirU instruments, considerable synchronicity between the GRIMM PM_2.5_ aerosol measurements and the adjusted AirU PM_2.5_ aerosol measurements was observed; i.e., for AirU 124, 106/133 (79.7%) of GRIMM and AirU measurements were both <5 μg/m^3^ when comparing the instrument’s measurement data recorded at the same time (with the same time/date stamp). Similarly, AirU 125 had 63/72 (87.5%) of measurements when both were < 5 μg/m^3^. Lastly, AirU 127 had 90/117 (76.9%) of measurements when both were < 5 μg/m^3^.

Further analysis with this adjusted data revealed that for AirU 124, 108/132 (81.2%) of measurements were within ±1 μg/m^3^ of the GRIMM measurement. AirU 125 had 69/72 (95.8%) of measurements within ±1 μg/m^3^ of the GRIMM. Finally, AirU 127 had 102/117 (87.2%) measurements within ±1 μg/m^3^ of the GRIMM. See Table 3 for a comparison between each AirU and the GRIMM.

Moving average calibration models (see Figure S2) between the months of November to March indicated a change in Y-intercept, slope, and R^2^ over time. These charts show that over the course of the field validation, there were changes in the Y-intercept, slope, and R^2^. However, both the slope and R^2^ were relatively stable over this study period, which agrees with the findings of the pre- and post-calibration tests using a laboratory calibration chamber. While the graphs shown in Figure S2 are not of optimal quality, the reader should keep in mind that they are included to just show variability and general changes in Y-intercept, slope, and R^2^ over time.

## 4. Discussion

Overall, findings from this study suggest that sensor degradation is possible among low-cost sensors used continuously for multiple months, which has been found by other researchers [35,43,45]. The pre- and post-equation laboratory comparisons suggested a gradual loss in the AirU’s precision and accuracy from the baseline calibration. However, for the deployment duration investigated here, these changes were overall minimal and not necessarily relevant for practical applications.

These findings were reinforced in the field comparison of the AirU to the GRIMM. The individual AirUs demonstrated a similar slight decrease in accuracy and precision between deployment and the end of the field validation. However, results indicate that at least 81.2% of the measurements from the AirU were within ±1 μg/m^3^ of the GRIMM. This suggests that given its cost-effectiveness and user-friendly design, the AirU remains a useful exposure assessment tool with only minimal sensor degradation over time.

### 4.1. Pre- and Post-Calibration Equations

Close examination of the pre- and post-equation comparisons revealed average percentage changes among the R^2^ to be 1–3% and Y-intercept to be ~300–600%. These were the two components that were statistically significantly different, while the slope, with an average percentage change of ~10–20%, was found to not be statistically significantly different over the course of this study. During the deployment period of January 2018 to October 2018, even though the R^2^ was statistically different, it is likely not meaningful in practice because the values were still greater than 0.9, which indicates a strong relationship. With the slope not being statistically different, but with the Y-intercept changed by over 300%, this suggests that the calibration has shifted upwards. However, it should be noted that all parameters of the linear regression calibration equation should be considered together to provide an understanding of calibration changes over time. The separation of the calibration equation into individual parameters, which are then analyzed independently, may not provide an accurate picture of sensor degradation. Another related important finding was that increased time in the field did not correspond to significant changes in absolute values of pre- and post-calibration equation parameters. In essence, while the average percentage change in Y-intercept was substantial, in terms of absolute measurements, the AirU did not demonstrate changes that would invalidate their measurements for most practical uses. This was demonstrated and reinforced by the field validation, in which the majority of AirU values were within ±1 μg/m^3^ of the “true value” (i.e., the GRIMM). In practice, this discrepancy does not present a meaningful difference for typical field-based exposure assessments.

### 4.2. Field Validation with GRIMM and AirUs

For household exposure assessment, the results found here indicate that the AirU is able to develop a useful characterization of air pollution. For the purposes of this type of work, an absolute change, as opposed to a percentage change, is arguably a more meaningful measure. However, it should be noted that without the linear regression model applied, the raw AirU data showed an underestimation for the lowest concentrations; a finding that was perhaps unsurprising due to previous findings that the LOQ for the AirU was approximately 5 μg/m^3^ [35,42]. Issues with the LOQ suggest that perhaps the AirU is more ideally suited for monitoring air pollution episodes, which have high PM concentrations, rather than day-to-day fluctuations in the home, which typically have lower PM concentrations unless a calibration curve is applied. Additionally, other long-term fields and laboratory evaluations of Plantower sensors have found both overestimation and underestimation, suggesting that constant calibration may be key to its effective use [42].

Previous studies have suggested that perhaps sensor variation was due to dust accumulation on the laser used for particle measurement [35]. The field comparison of the AirUs and GRIMM carried out in this work included maximum PM concentration levels of approximately 16 μg/m^3^ for the GRIMM and approximately 60 μg/m^3^ for the AirU and average PM concentration levels of 2.8 μg/m^3^ for the GRIMM and approximately 8.6 μg/m^3^ for the AirU. These low PM concentration levels indicate that dust accumulation may not be the primary cause of sensor variation. These findings indicate that sensor variation, although small, exists, and can occur even in low indoor PM concentration conditions, as opposed to previous studies that have recorded variation in outdoor evaluations [42]. Another study measuring sensor variation in the Dylos DC1100 low-cost particle counter found measurement variation even after the sensor was cleaned, indicating either a cleaning method issue or sensor degradation via hardware changes from continuous usage [36].

### 4.3. Limitations

In spite of existing knowledge that the AirUs may report negative concentrations (−1.0 μg/m^3^) under certain hardware situations, as well as an update to all circuit boards that took place prior to this study, this continued to affect measurements in this study. Another design flaw of the AirU was its current lack of direct reading capability to immediately report data, which prevented the immediate realization that the SD card was loose, resulting in the loss of a month of data. During the course of this project, secure wireless internet connectivity and a web-based and mobile application have been developed to provide real-time measurement in this device. The GRIMM instrument posed some technical difficulties as well; in spite of typically reliable reporting, the GRIMM randomly skipped minutes or hours of data at a time. This is to be noted for future research that requires continuous measurements using the GRIMM.

Ambient conditions also posed some limitations to the study, particularly humidity readings. Higher relative humidity is associated with higher PM_2.5_ concentrations ([46]. In the case of the field comparison, relative humidity stayed low throughout the time range, with a maximum humidity percentage of ~25%. However, because it was relatively dry (<25%) throughout the field test, electrostatic discharge was more likely to have occurred, which could have affected overall PM levels (Manufacturing 2014).

### 4.4. Recommendations for Future Work

With the growing proliferation of low-cost sensors, it is critical to test sensors in a number of different environments and contexts to determine the limits of sensor capabilities. Since the AirU has proven to be a reliable tool in this and previous studies, future research should continue to determine the conditions in these sensors are most accurate and reliable. In particular, future work should seek to validate the usefulness of the AirU in indoor and outdoor settings. A previous study on indoor air quality found that there were low concentrations of particulates despite being a fully occupied office [47]. This raises questions about the particle of choice used in calibration. Arizona road dust or ISO test dust has been frequently used in aerosol research and may be more representative of and can simulate what is seen in indoor air pollution [48,49]. Therefore, if AirU sensors are to be used to assess indoor air in future studies, it is recommended to use Arizona road dust or ISO test dust in instrument calibration, rather than ammonium nitrate, because it is more relevant for indoor air and has been shown to provide adequate calibrations for other low-cost sensors [26]. Findings from this study and previous research indicate that sensor variation was likely, although changes might not be due to dust accumulation, as has been previously posited. A future study based on dust accumulation is therefore suggested to further investigate this finding.

Another recommendation for future work is the development of a direct-reading tool for the AirU, which is currently in the process of implementation. A smartphone application designed to report readings directly from the AirU to the user is due to be released in 2020. With this application, homeowners and/or citizen scientists can closely track their own exposures and be more actively involved in their own exposure assessment. In addition, researchers would be able to increase the quality of research by immediately addressing missing data or malfunctioning equipment.

## 5. Conclusions

This study examined AirU sensor measurement degradation over deployment periods of up to six months. The absolute concentration values, both measured over time and in comparison with the GRIMM, suggest that while sensor variation does exist, it was likely not large enough to result in meaningful differences in practical applications. Economically, an AirU sensor costs orders of magnitube less than reference instruments (approximately 250 USD at the time of this writing). This places the AirU in the unique position of being economically viable to individuals interested in monitoring their indoor air quality and for mass deployments for environmental epidemiological studies investigating the relationship between indoor air quality and health outcomes. The AirU is therefore still recommended as a useful low-cost particle sensor for use in air quality research. However, additional research is needed to fully understand the performance capabilities and environments in which to use the AirU. This may necessitate a change in calibration procedures and an experiment focusing on dust accumulation to better understand the limits of this device.

## Figures and Tables

**Table 1:** Changes in calibration equations among AirUs deployed in Project ECHO.

Time in Field	Avg. Slope % Difference	Avg. Y-Intercept % Difference	Avg. R^2^ % Difference
<3 months (n = 6)	14%	433%	1%
3–5 months (n = 13)	18%	608%	3%
>5 months (n = 5)	11%	512%	1%

**Table 2:** Descriptive statistics for AirU and GRIMM comparison study (all values in μg/m^3^).

	AirU 124	AirU 125	AirU 127	GRIMM
Min	0.04	0.01	0.03	0.23
Max	58.86	57.37	61.93	16.89
Mean	11.38	6.14	9.88	2.82
Median	5.15	2.05	4.61	1.87

**Table 3:** The GRIMM analyzed against the AirUs for a −1 μg/m^3^ and +1 μg/m^3^ change in concentration measurement. This chart shows the number of observations in which the AirU and the GRIMM agreed.

	AirU 124	AirU 125	AirU 127
GRIMM value within ±1 μg/m^3^ of AirU	108 (81.2%)	69 (95.8%)	102 (87.2%)
GRIMM value outside ±1 μg/m^3^ of AirU	24	3	15

## Data Availability

Not applicable.

## Supplementary Materials

The following supporting information can be downloaded at: https://www.mdpi.com/article/10.3390/toxics11010056/s1, Figure S1: Scatterplot comparing AirU daily averages (in µg/m^3^) to daily Grimm measurements, showing each linear regression line and its associated R^2^. Figure S2. Changes in AirU/Grimm calibration elements over the study period for (a) AirU124, (b) AirU 125, and (c) AirU 127.
