# Supplementary material for: Aerosol Measurement Degradation in Low-Cost Particle Sensors Using Laboratory Calibration and Field Validation
Source: Toxics. 2023 Jan 6;11(1):56. doi: 10.3390/toxics11010056 (PMC9862639; doi:10.3390/toxics11010056)
Supplement: Supplementary file 1 [file toxics-11-00056-s001.zip › toxics-1993614-supplementary.pdf]

a) AirU 124 $R^2 = 0.82$

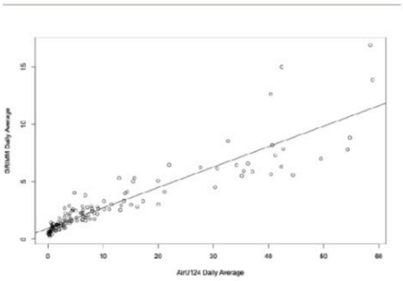

b) AirU 125 $R^2 = 0.97$

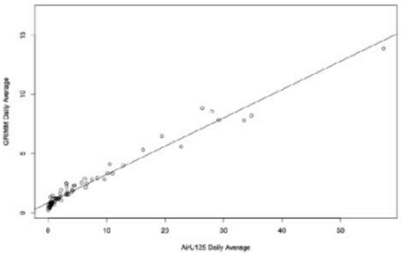

c) AirU 127 $R^2 = 0.935$

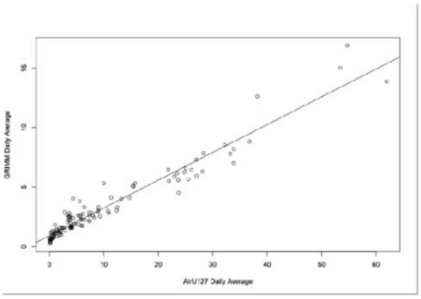

Figure S1. Scatterplot comparing AirU daily averages (in μg/m³) to daily Grimm measurements, showing each linear regression line and its associated R^2.

a)

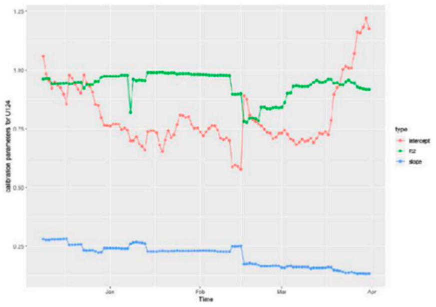

b)

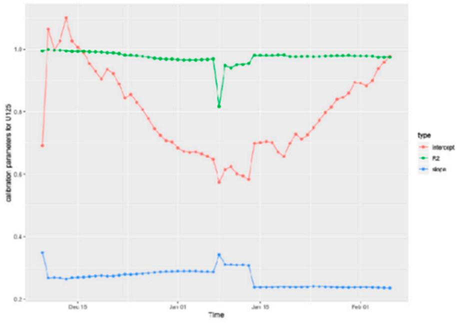

c)

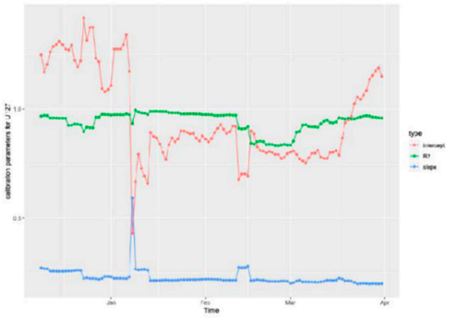

Figure S2. Changes in AirU/Grimm calibration elements over the study period for (a) AirU124, (b) AirU 125, and (c) AirU 127.
